# The effects of extreme heat on human health in tropical Africa

**DOI:** 10.1007/s00484-024-02650-4

**Published:** 2024-03-25

**Authors:** Joshua Jonah Kunda, Simon N. Gosling, Giles M. Foody

**Affiliations:** School of Geography, University of Nottingham, University Park Nottingham, NG7 2RD, UK

**Keywords:** Climate change. Extreme heat. Human health. Tropical Africa

## Abstract

This review examines high-quality research evidence that synthesises the effects of extreme heat on human health in tropical Africa. Web of Science (WoS) was used to identify research articles on the effects extreme heat, humidity, Wet-bulb Globe Temperature (WBGT), apparent temperature, wind, Heat Index, Humidex, Universal Thermal Climate Index (UTCI), heatwave, high temperature and hot climate on human health, human comfort, heat stress, heat rashes, and heat-related morbidity and mortality. A total of 5, 735 articles were initially identified, which were reduced to 100 based on a set of inclusion and exclusion criteria. The review discovered that temperatures up to 60°C have been recorded in the region and that extreme heat has many adverse effects on human health, such as worsening mental health in low-income adults, increasing the likelihood of miscarriage, and adverse effects on well-being and safety, psychological behaviour, efficiency, and social comfort of outdoor workers who spend long hours performing manual labour. Extreme heat raises the risk of death from heat-related disease, necessitating preventative measures such as adaptation methods to mitigate the adverse effects on vulnerable populations during hot weather. This study highlights the social inequalities in heat exposure and adverse health outcomes.

**Supplementary Information:**

The online version contains supplementary material available at 10.1007/s00484-024-02650-4.

## Introduction

Most studies on heat-human health have focused on high income countries (HICs) compared to low- and middle-income countries (LMICs), for which few studies have been conducted, particularly in tropical Africa (Basu 2009; Green et al. 2019; Ebi et al. 2021; Kotharkar and Ghosh 2022). High temperatures can cause a rise in core body temperature and heart rate and lead to heat stress, heat stroke and, in extreme cases, death. Individuals with heart disease, obesity, or respiratory conditions are more vulnerable to heat stress (Donaldson et al. 2003; Kenney et al. 2014; Rahman and Adnan 2023). Among the effects of high temperatures on human health are heat exhaustion, dehydration, respiratory issues, cardiovascular strain, skin diseases, mental health issues, and electrolyte imbalance (Basu and Samet 2002; Gosling et al. 2009; Hajat and Kosatky 2010; Gabriel and Endlicher 2011; Hondula et al. 2012; Ma et al. 2014; Alcoforado et al. 2015; Son et al. 2016; Mora et al. 2017). High temperatures not only exacerbate existing heat-related health conditions, leading to organ failure and mortality, but also cause a range of harmful effects such as an increase in violent crimes (Sanz-Barbero et al. 2018), fatal road accidents (Wu et al. 2018), and stress on ambulance services (Dolney and Sheridan 2006; Cheng et al. 2016; Guo 2017). Rising temperatures also increase electricity and water demand (Hatvani-Kovacs et al. 2016), impacting infrastructure, water quality, open spaces, and overall liveability in urban areas (Klok and Kluck 2018).

The effects of heat on human health are further exacerbated by environmental, socioeconomic, demographic, physiological and behavioural factors. For instance, urban areas with high population density, limited green space, and extensive artificial impervious surfaces (AIS) can be warmer than surrounding areas (Myint et al. 2013; Chen et al. 2022a, b; Rajagopal et al. 2023). Economic constraints can limit access to cooling systems, adequate hydration, and healthcare services, e.g. inadequate access to air conditioning and other cooling methods can increase human vulnerability during extreme heat. Living in poorly ventilated homes can exacerbate the harmful effects of extreme heat (Thomson et al. 2019). Limited access to healthcare can hinder the treatment of heat-related illnesses; the effectiveness of public health interventions and heatwave warning systems plays a crucial role in exacerbating the harmful effects of extreme heat (Foster et al. 2020; Périard et al. 2021; Hess et al. 2023). Outdoor workers, such as those in construction, mining, and agriculture, are more exposed to high temperatures (Jay et al. 2021; He et al. 2023). Isolated individuals may lack assistance during heat waves (Kenny et al. 2020; Habibi et al. 2023). Older populations and young children are more sensitive to heat due to less effective thermoregulation (Tsuzuki 2023). A lack of awareness of heat risks can lead to inadequate preventive measures (Jessel et al. 2019). Cultural norms and practices, for instance, clothing choices, might affect how individuals respond to heat (Sovacool et al. 2021).

The occurrence of hot days in tropical Africa has been increasing since the 1980s because of increasing greenhouse gas (GHG) emissions, which continue to alter the region's summer temperatures (Mahlstein et al. 2011; Harrington et al. 2017; Herold et al. 2017; Ntoumos et al. 2022). Temperatures in tropical Africa are frequently near the upper limit of human comfort (Sherwood and Huber 2010). For example, in 2010, extreme temperature incidents of 47.6 °C and 48.2 °C were recorded in Faya-Largeau, Chad Republic, and Bilma, Niger Republic, respectively (World Meteorological Organization 2016). Furthermore, between 1989 and 2009, tropical Africa recorded 40 to 50 heat waves annually (Cook and Vizy 2012; Iyakaremye et al. 2021). The Nigerian Meteorological Agency (2021) recently reported extreme heatwave events of 50 °C in the Northern-eastern region of Nigeria. The Emergency event database (EM-DAT 2023) reports an incident with an extreme temperature of 60 °C in Nigeria which killed 60 people. (Table 1).

**Table 1:** List of heat events by region and country and the total human morbidity and mortality associated with each event

Sources	Country	Date/year	Tmax (^o^C) recorded	Number of people affected /Total mortality
EM-DAT (2023)	Nigeria	June 2002	60°C	60
	South Africa	January 2016	45°C	11
	Sudan	August 2015	47°C	16
	Algeria	01 July 2003	47°C	40
Kynast-Wolf et al (2006)	Burkina Faso	1993–2001	Not mentioned	4, 098
Africanews (25/01/2023)	South Africa	24/01/2023	40°C	8
Arsht-Rock’s new report 7 August 2023	Nigeria	Not mentioned	Not mentioned	17,000 excess heat wave deaths, of which half are women
Heat health information network (2013)	Senegal	May, 2013	45°C	A reported mortality increase (observed by medical staff) of 12.4%
Kynast-Wolf et al (2010)	Burkina Faso	1999–2003	40°C	1238
Bunker et al (2017)	Nouna, Burkina Faso	2000–2015	43.9°C	790 NCD deaths, corresponding to 18,367 years of life lost (YLL)
Arisco et al (2023)	Burkina Faso, Nouna region	2000–2005	41.1°C	Out of the 8256 total deaths, 6185 were caused by climate –sensitive diseases
Ye et al (2009)	Nairobi, Kenya	2003–2005	Not mentioned	436 deaths observed in children under 5 years, with pneumonia as the leading cause
Egondi et al (2012)	Nairobi, Kenya	2003–2012	38.2°C	4,671 all causes of death
Faye et al (2021)	Banda Fassi, Senegal	1973–2012	49°C	Not specified
Azongo et al (2012)	Kassena-Nankana, Ghana	1995–2010	44.2°C	Not specified
Wright et al (2017a, b)	Limpopo province of South Africa	2017	For every 1°C increase in average daily temperature	6% increase in hospital admissions for diarrhoea among individuals of all ages, and a 4% increase in admissions for individuals older than 5 years
Bonell et al (2022)	West Kiang region, The Gambia	2019—2020	Tmax: 33.5°C WBGT: 35.1°C UTCI: 51.3°C	3 cases of stillbirth or intrapartum death among the participants
Bühler et al (2022)	Limpopo Province, South Africa	2009—2016	32°C	9.5% of cardiovascular disease (CVD) admissions were attributable to non-optimal temperatures (cold and warm combined), with 8.5% attributable to cold temperatures and 1.16% to warm temperatures
Thompson et al (2012)	Mussina, Limpopo province of South Africa	1999—2010	32.2°C annual increase of 0.15°C	4.0% of children admitted for heat-related diseases died
Arisco et al (2023)	Nouna, Burkina Fasso	2000—2015	47.2°C	Of the total 8,256 deaths recorded, 6,185 (74.9%) were due to climate-sensitive diseases
Nyadanu et al (2023)	Ghana	2012—2020	UTCI 28.8°C	Linked UTCI with 90,532 stillbirths out of 5,961,328 births
Étard et al. (2004)	Senegal	1989—2000	Not mentioned	Diarrhoeal diseases, malaria, and acute respiratory infections account for 3,424 deaths before the age of 15
Mutisya et al (2010)	Nairobi, Kenya	2003—2005	34°C	436 deaths among children under five, translating to an overall death rate of 19.95 per 1,000 person-years
Luque Fernández et al. (2009)	Lusaka, Zambia	2003—2006	36°C	1°C increase in temperature 6 weeks before the onset of the outbreak was associated with a 5.2% increase in the number of cholera cases
Jaffar et al (1997)	Upper river division, Gambia	1989—1993	36°C	80.1 per 1,000 for infants and 18.8 per 1,000 for children aged 1–4 years

The effects of extreme heat on human health in LMICs are often exacerbated by socioeconomic and demographic characteristics of the population, for example, poverty, literacy, infants, and aged population (Oluwafemi et al. 2023; Nyadanu et al. 2023). The IPCC (2022) reported that LMICs had limited adaptive capacity to extreme heat due to scarce resources, fragile political institutions, and socio-cultural practices. More research on heat-human health in tropical Africa is needed (Omonijo et al. 2013; Agan 2017; Leal Filho et al. 2018; Niu et al. 2021) to identify the requirement for enhancing the resilience of the region to climate change-enhanced extreme heat events. Recent studies indicate that some HICs have observed a decline in the sensitivity of health outcomes to extreme heat, which implies an increase in adaptive capacity to extreme heat (Coates et al. 2014; Bobb et al. 2014; Sheridan and Allen 2018; Laranjeira et al. 2021). Unlike HICs, vulnerability to extreme heat in LMICs is on the rise due to their increase in sensitivity and low adaptive capacity to extreme heat (Hajat et al. 2010; Azhar et al. 2017; Green et al. 2019; Ncongwane et al. 2021; Chen et al. 2022a, b). This review aims to summarise the peer-reviewed literature on the relationship between extreme heat and human health in tropical Africa.

## Material and methods

### The regional focus of the review

**Fig. 1:**
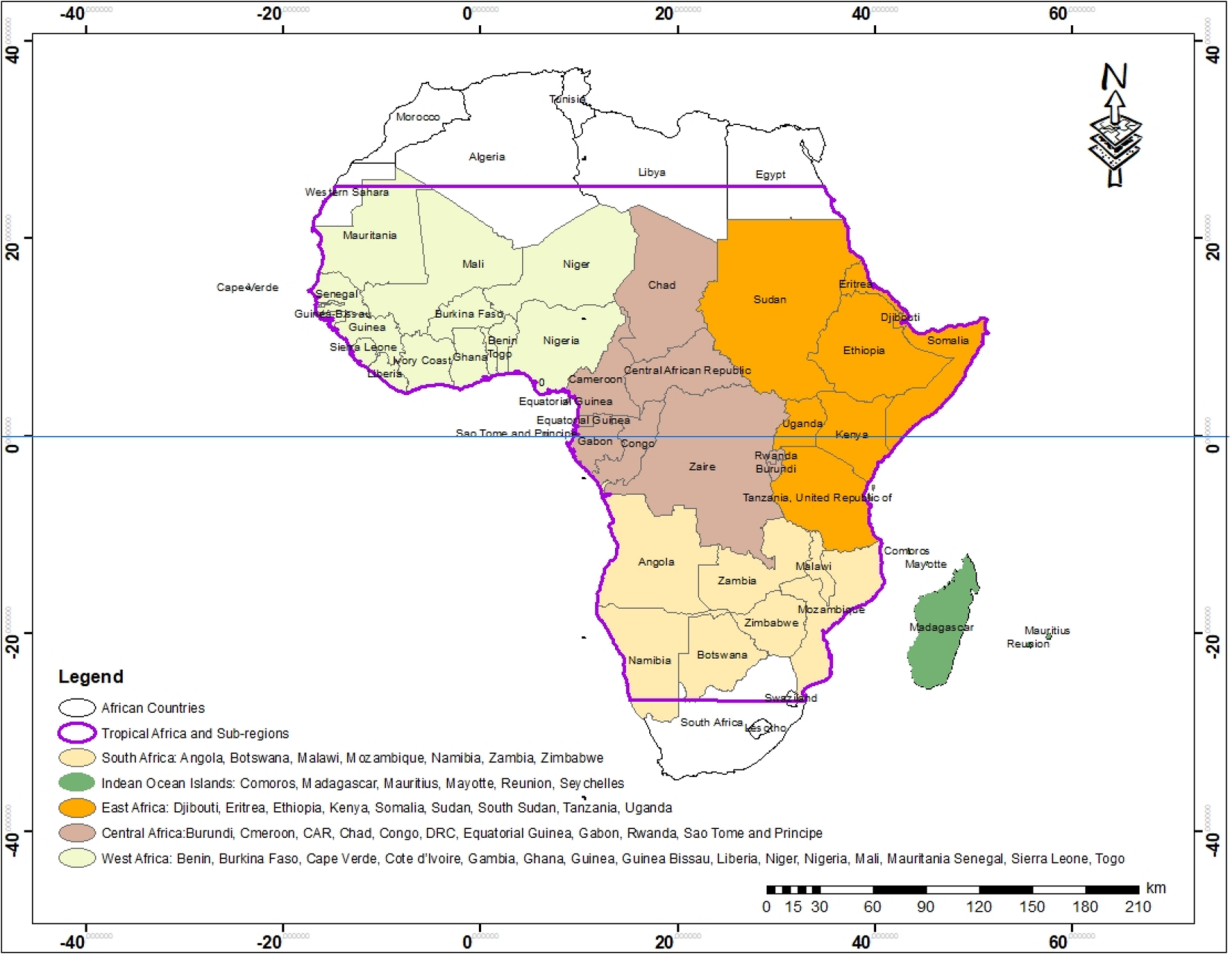
The tropical Africa region considered in this review

Tropical regions lie between latitudes 23.5° north and south of the equator. Tropical Africa consists of 48 countries that make up five sub-regions: West Africa (16 countries), East Africa (9), Central Africa (10), part of Southern Africa (7), the Indian Ocean islands, and Madagascar (6). Figure 1 presents the sub-regions and member countries of tropical Africa. The Köppen-Geiger system classified the climate of tropical Africa as Type ‘A,’ characterised by constant, elevated temperatures and generally humid (Af) with high amounts of precipitation because of their closeness to the equator (Burkart et al. 2014). However, there is the emergence of drier climatic areas with declining rainfall towards latitude 23.5° north and south away from the equator due to the influence of the subtropical high-pressure system (Aw) and transition from type ‘A’ to type ‘B’ arid climates. Generally, regions at high altitudes have lower temperatures, typical of warm temperate-type ‘C’ climates (Kottek et al. 2006).

The weather and climate of tropical Africa varies with geographical location and is influenced by topography, proximity to large water bodies and movements of the Intertropical Convergence Zone (ITCZ; Odekunle et al. 2005; Oluwafemi et al. 2023). There are two major seasons in tropical Africa: rainy and dry seasons. The rainy season in Central and West Africa, e.g. Nigeria and Congo, start from April to October, with annual rainfall of 1,000—2,500 mm. The dry season lasts from November to March (Adeniyi and Oyekola 2017; Odekunle et al. 2005; Adegebo 2022). East Africa, e.g., Kenya and Ethiopia, is characterised by two rainy seasons—the long rainy season from March to May and the short rainy season from October to December, with an average rainfall of 500—1,500 mm. The dry season in this region occurs between the two rainy seasons and after the short rains (Camberlin and Philippon 2002; Cattani et al. 2018). Generally, the dry season is characterised by lower humidity, less cloud cover, and little or no rainfall. Tropical Africa generally experiences a warm climate, with temperatures ranging from 25°C to 30°C (Odekunle et al. 2005). However, there are temperature variations; for instance, highlands, e.g., Ethiopian highlands, have lower temperatures, below 20°C (Camberlin and Philippon 2002). Coastal regions have more consistent temperatures, influenced by oceanic currents, with an average monthly temperature of 31◦C and 32◦C in February and March and reaching their lowest temperature of 27◦C to 28◦C in July and August (Oluwafemi et al. 2023). The Sahelian Region at the northern fringes can experience more extreme temperatures over 40°C in March and April due to its proximity to the Sahara Desert (Agada and Yakubu 2022).

### Search approach

Literature searches were performed in the Web of Science (WoS) to identify research articles on the association between extreme heat and human health in tropical Africa. The search terms were narrowed to peer-reviewed articles written in English. 5,735 publications were initially identified. Searches included all publications in the WoS database up to and including December 2023. Table 2 shows the keywords and search terms that were used to search “All fields” in the WoS database, which included health outcomes that are commonly referred to in heat health studies (e.g. human health, heat-related mortality) and several climatic and biometeorological climate variables that broadly cover the totality of the effect of weather and climate associated with extreme heat on temperature-related health by accounting for temperature, humidity, wind speed and radiation (e.g. high temperature, Wet Bulb Globe Temperature (WBGT), Universal Thermal Climate Index (UTCI); see Gosling et al. (2014) for definitions).

**Table 2:** Keywords search terms across all fields in the WoS database

	Keywords search term	Results from the search terms (WoS)
1	(extreme heat OR humidity OR WBGT OR apparent temperature OR wind OR Heat Index OR Humidex OR UTCI OR heatwave OR high temperature OR hot climate) AND "heat stress."	316
2	*(extreme heat OR humidity OR WBGT OR apparent temperature OR wind OR Heat Index OR Humidex OR UTCI OR heatwave OR high temperature OR hot climate) AND “human* health*."*	1, 688
3	*(extreme heat OR humidity OR WBGT OR apparent temperature OR wind OR Heat Index OR Humidex OR UTCI OR heatwave OR high temperature OR hot climate) AND “heat* related mortality.*"*	396
4	*(extreme heat OR humidity OR WBGT OR apparent temperature OR wind OR Heat Index OR Humidex OR UTCI OR heatwave OR high temperature OR hot climate) AND “heat* related morbidity.*"*	943
5	*(extreme heat OR humidity OR WBGT OR apparent temperature OR wind OR Heat Index OR Humidex OR UTCI OR heatwave OR high temperature OR hot climate) AND “thermal* comfort.*"*	2176
6	*(extreme heat OR humidity OR WBGT OR apparent temperature OR wind OR Heat Index OR Humidex OR UTCI OR heatwave OR high temperature OR hot climate) AND “heat* rashes.*"*	216

A preliminary scan of the articles identified after conducting the searches listed in Table 2 was undertaken to eliminate studies that examine non-human impacts, such as those on plants and animals. A manual check on the articles' titles, abstracts, and main text was undertaken for further screening using the inclusion criteria below:

1. Studies carried out in any part of a country located between the tropics in tropical Africa, which focused on the effects on human health from increasing temperature, extreme heat, or heatwaves, and considered humidity, wind speed, solar radiation, or hot climate.
2. Studies carried out in any part of a country located between the tropics in tropical Africa, which have considered the effects of heat as modifiers of deaths/infections from malaria, Trypanosomiasis, Schistosomiasis, and other infectious diseases.

**Fig. 2:**
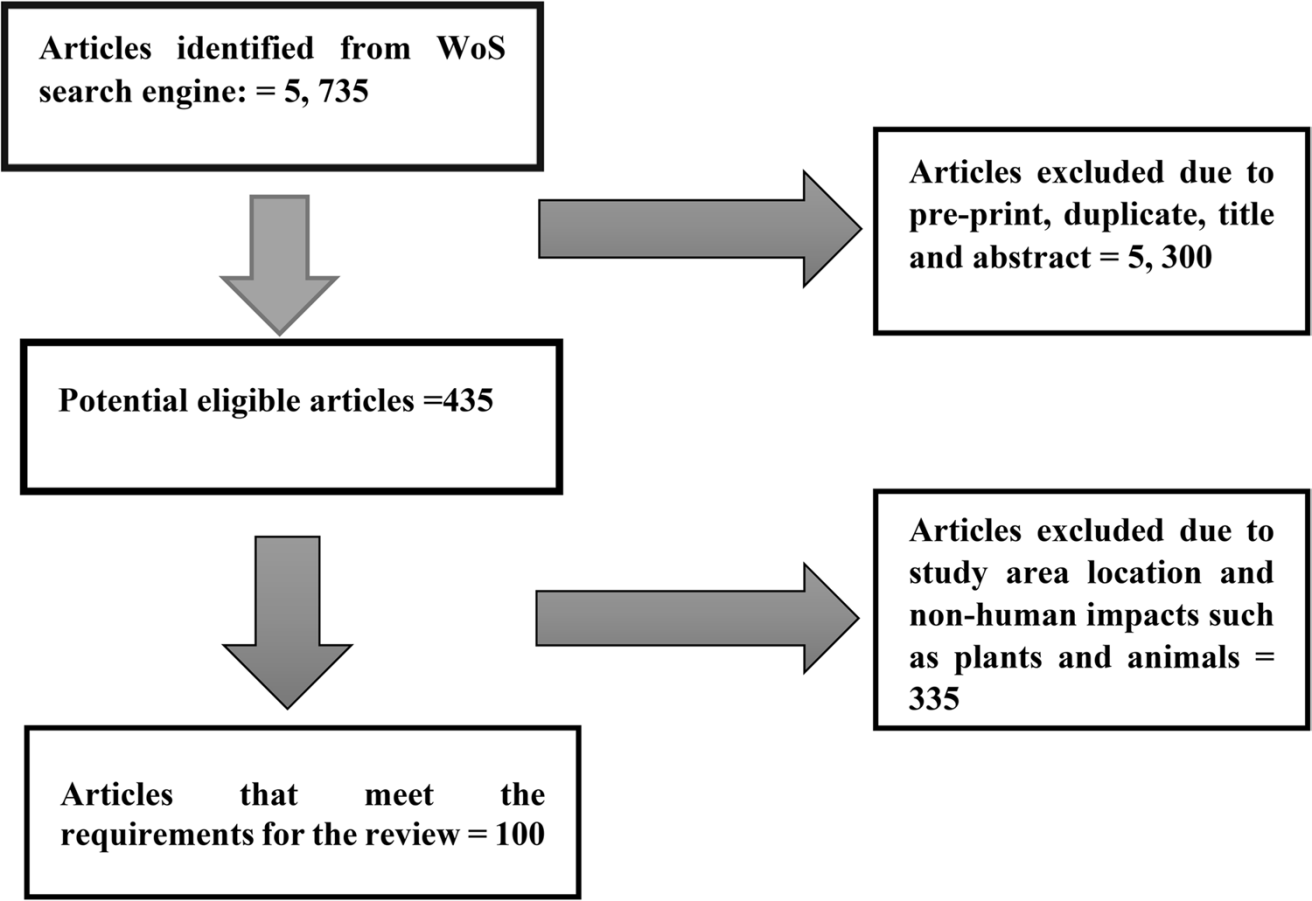
The literature search procedure

After removing duplicate entries and articles due to study area location and the 2 inclusion criteria, 100 articles met the requirement for this review as shown in Fig. 2.

### Studies identified by the review

The number of studies on the effects of heat on human health, organised by country in tropical Africa, is shown in Table 3. Many studies have been conducted in Nigeria, Ghana, Kenya, Tanzania and Burkina Faso, Gambia, and South Africa. Only one or two studies have been published for many countries. There were no studies done in the following 17 tropical African countries: Niger, Chad, Mauritania, Ethiopia, Somalia, Eritrea, Togo, Ivory Coast, Liberia, Sierra Leone, Guinea Bissau, CAR, Zaire, Tanzania, Angola, Namibia, and Batswana. There have also been no studies conducted in African Countries that are partially in the tropics, such as Algeria (Tamanrasset Province), Egypt (Aswan Governorate), Libya (Al Kufra), and Western Sahara. 9(9%) studies are multi-country, encompassing a wide range of environments, socioeconomic and populations, 5(5%) of which were conducted in West Africa and 1(1%) in Central Africa. 1(1%) study was conducted in Kenya, Zambia, and Zimbabwe; Guinea, Gabon, the Democratic Republic of the Congo, South Sudan, and Uganda; and Kenya, Uganda, Rwanda, Burundi, Tanzania, Zambia, Malawi, and Mozambique. A multi-country study offers a more reliable understanding of tropical Africa's complex interactions between heat and human health.

**Table 3:** The number of studies on the effects of heat on human health, organised by country

Study location	Number of studies
Nigeria	22
Ghana	13
Kenya	9
Tanzania	8
Burkina Faso	7
Gambia	6
South Africa	14
Senegal	4
Zambia	3
Zimbabwe	3
Uganda	3
Democratic republic of Congo	2
Cameroon	2
Mozambique	1
Malawi	1
Burundi	1
Rwanda	1
Sudan	1
Mali	1
Gabon	1
Guinea	1
Benin	1
West Africa	5
Central Africa	1
Kenya, Zambia, and Zimbabwe	1
Guinea, Gabon, DRC, South Sudan, Uganda	1
Kenya, Uganda, Rwanda, Burundi, Tanzania, Zambia, Malawi, Mozambique	1

### The temporal resolution of studies and the length of time that they explored health impacts

The temporal resolution of the studies relates to whether the data was collected daily, weekly, monthly, seasonal, annually, and future projections. The length of the study relates to how long the studies investigate health impacts, i.e., how many days or years of data were used for the study.

Concerning the temporal resolution of the data, 15 (15%) of the studies project the future effects of heat on human health (Lorena et al. 2018; Ragatoa et al. 2018; Fotso-Nguemo et al. 2022). 25 (25%) studies used hourly, daily, monthly, and seasonal datasets, e.g. Azongo et al (2012) and Faye et al (2021) studied heat exposure on a daily scale. Brewster and Greenwood (1993) and Frimpong et al (2014) explored seasonal scale variations. 43 (43%) of the studies are based on annual and multi-annual scales. These were heat-health studies lasting years or decades. For example, studies by Etard et al (2004) and Fotso-Nguemo et al (2022) cover 11 and 39 years, respectively.

It may be argued that studies founded on annual and multi-annual scales, as opposed to daily, weekly, monthly, or seasonal studies offer a better extrapolation of the association between extreme heat and human health because it enables a more accurate assessment of the effects and changes over time. This depends on whether the study is a clinical trial assessing the immediate effects of excessive heat over a relatively short period or a cohort study exploring the long-term health effects of extreme heat to monitor the change over time. For instance, case-crossover studies are a type of observational research design commonly used in epidemiology and public health to investigate the association between an exposure such as heat exposure and an outcome such as a health outcome, these studies are beneficial for studying the acute effects of transient exposures on short-term outcomes.

### Methods and technologies for data collection and analysis

Systematic data collection from weather stations is often used (Olatunde 2016; Azongo et al. 2012; Luque Fernández et al. 2009) while other studies use data from remote sensing, for example Wiru et al (2020), Mutai (2013), and Paz (2009) used satellite data from the National Climate Data Centre of the National Oceanic and Atmospheric Administration. Herold et al (2017). Balogun and Balogun (2014), Kwasi et al (2014), and Balogun and Daramola (2019) used a Shielded portable Lascar EL-USB-2 data logger for collecting observed temperature and relative humidity data. Some experiments utilised technological data observations concerning the data type, such as temperature and relative humidity, from a weather station or a Shielded portable Lascar EL-USB-2 data recorder (Adeniyi 2009; Frimpong et al. 2016; Balogun and Daramola 2019). Due to their cost, portability, and convenience, the use of Lascar USB temperature and humidity sensors with a calibrated Questemp heat stress monitor for daily, monthly of seasonal studies has increased in recent years (Balogun and Balogun 2014; Kwasi et al. 2014; Frimpong et al. 2016). Other research methods include questionnaires, surveys, and FGD (Ngwenya et al. 2018; Frimpong et al. 2020; Nunfam 2021). Nevertheless, some studies rely on hospital health data (Etard et al. 2004; Diboulo et al. 2012; Wiru et al. 2020).

13 (13%) of the articles cited in this review utilised reanalysis and climate models to simulate past, present, and future heat-human health relationships. 5 (5%) of studies explored future projections of heat stress, high temperature, relative humidity, heatwaves, and extreme heat on human health (Ermert et al. 2012; Sylla et al. 2018; Sarr et al. 2019; Gyilbag et al. 2021; Ragatoa et al. 2018). Reanalysis and climate models provide spatially gridded, historical and future climatic data, essential for studying long-term trends and potential future scenarios of heat impacts on human health across large spatial domains, aiding public health planning and climate change adaptation strategies. The output from these models often contains uncertainties due to assumptions and limitations in data and might not accurately capture local variations, leading to less precise assessments at local scales. 6 (6%) of the cited studies are at the regional scale, e.g. (Blom et al. 2022, Adeniyi and Oyekola 2017, Sylla et al. 2018, Batté et al. 2018; Ermert et al. 2012) covered West Africa, whereas Fotso-Nguemo et al. (2022) covered central Africa.

Several studies have used high resolution regional climate model simulations to estimate the effects of different greenhouse gas emissions scenarios on future health in tropical Africa. Some studies have used climate projections from the recent Coordinated Regional Climate Downscaling Experiment (CORDEX) program (Sarr et al. (2019), Ragatoa et al. (2018), Sylla et al. (2018), Gyilbag et al. 2021) and Adeniyi and Oyekola (2017)), for either Representative Concentration Pathway (RCP) greenhouse gas scenarios or global warming scenarios. Other studies have used the COSMO-CLM regional climate model, e.g. Ermert et al. (2012) and Fotso-Nguemo et al. (2022) considered a 1.5 °C global warming scenario. Diouf et al. (2013) used two, older, SRES emissions scenarios. No studies to date have considered the latest SSP (Shared Socioeconomic Pathways) scenarios and/or simulations from CMIP6 climate models (Coupled Model Intercomparison Project).

Over 47 (47%) of the identified studies employed descriptive and inferential statistics to analyse daily, monthly, and seasonal data from field surveys, FGD, questionnaires, and interviews (Alaigba et al. 2018; Ngwenya et al. 2018; Nunfam 2021). Annual and multi-annual studies such as 30 years, frequently employ time series, regression, and correlation designs to directly compare health data with biometeorological factors (Scott et al. 2017; Asamoah et al. 2018; Wiru et al. 2020).

### Summary of the review findings

Table (Online resources 1) summarises studies on the effects of extreme heat on human health in tropical Africa. Even though most studies identified showed an increase in morbidity and mortality in the hot/rainy season compared to the cool season (e.g., Kynast-Wolf et al. 2006; Mutisya et al. 2010; Diboulo et al. 2012; Scott et al. 2017), studies in Bono village of Ghana revealed an increased risk of death at the lowest Apparent Temperature (18°C). Specifically, the highest relative mortality risk (RR = 1.61, 95% CI: 1.21–2.15, *p*-value < 0.001) was observed three days after exposure to an apparent temperature of 18 °C, indicating a substantial increase in the risk of death compared to other apparent temperatures studied such as the first quartile (23 °C), third quartile (26 °C), and the highest apparent temperature (31 °C) that showed no significant relationship with mortality (Wiru et al 2020). In Botswana, Alexander et al (2013) found that minimum temperatures were related to increase Diarrhea occurrence. A study by Rayco-solon et al (2004) revealed seasonality in death rates, with more deaths occurring during the "hungry" season (July – November), marked by the peak of agricultural work, depletion of food supply, and a rise in infectious diseases. Diboulo et al (2012) noted a substantial increase in deaths with heat intensification at lags of 0 – 1 days. A temperature rise of 1.0°C at lag 0—1 was associated with a 2.6% increase in mortality for all ages and 3.7% for children under five years, with people over 60 most vulnerable to extreme heat. Asamoah et al (2018) found a 42% rise in the likelihood of suffering a miscarriage with every degree rise in Wet Bulb Globe Temperature (WBGT), suggesting a connection between atmospheric heat exposure and adverse pregnancy outcomes in Accra. It was also discovered that an increase in Temperature to over 40°C during summer could affect the population who spend long hours in the heat, such as street vendors (Ngwenya et al. 2018).

Frimpong et al (2020) found that heat stress considerably influences farmers in Bawku East of Northern Ghana, with malaria and heat cramps identified among the recurring diseases. Nunfam et al (2019a) established a relationship (*p* < *0.05*) between historical climate change threat awareness and work-related heat stress and the variance in educational accomplishment in the dissemination of coping approaches to work-related pressure from extreme temperatures. This agrees with the conclusions of Nunfam et al (2019b), who found a major variation in temperature-related morbidity with the type of mining activities among workforces in five mining spots in Western Ghana. Temperature and precipitation have altered the growth rates and survival of malaria pathogens. Several studies have demonstrated a decline in the spread of malaria in West Africa because of climate change-related increases in temperature and a decrease in precipitation (Ermert et al. 2012). In contrast, McGregor et al (1961), Lawoyin (2001), Reyburn et al (2011), and Ifatimehin and Ujoh (2014) observed a rise in morbidity or death during the rainy/hot season. Daniel (2015) reported a significant relationship between extreme temperature, rainfall, and heat rash.

### Socioeconomic factors that contribute to population vulnerability to heat

Three (3%) of the articles identified in this review examined socioeconomic characteristics that contribute to increasing population vulnerability to heat. Grace et al (2012) considered the influence of education, home water supply, floor material, and livelihood zones to explore the association between surface temperatures, rainfall, and stunting in children under 5 years. Ibu and Bisong (2021) explored the urban bioclimatic discomfort index in Calabar, Nigeria, using socioeconomic and demographic parameters such as the urban heat island effect, age sensitivity, biophysical and sociocultural data, urban planning, and health. The study emphasises the need to integrate age and urban environmental factors in measuring vulnerability to heat discomfort in cities. Oluwafemi et al. (2023) considered the urban heat island, population density, age and health conditions such as elderly, young children, people with chronic diseases or disabilities, and low-income populations that have less capacity to adapt, as well as living conditions of people in informal settlements and areas with less vegetation. The study identified critical heat risk zones covering approximately 423 km^2^ in in densely populated areas.

Including demographic and socioeconomic factors is crucial in heat-human health studies because different age groups, health statuses, and socioeconomic classes have different sensitivities to heat, affecting their health differently. High population densities, especially in urban areas, exacerbate the urban heat island effect, which increases health risks. Socioeconomic status influences access to cooling resources, healthcare, and information on extreme heat, which is essential for mitigating heat-related health risks. Understanding these factors aids in developing targeted strategies to protect the most vulnerable populations from heat-related health issues.

### Lag effects

The "lag period" refers to the time delay, often measured in days, between exposure to high temperatures and the observable health effects due to exposure. Lag periods vary between studies, e.g. 19 (19%) of the articles cited in this study observed a lag period of 0–28 days, 3 (3%) observed a lag period of 6–8 weeks, and 10 (10%) 1–10 months. 68 (68%) of the studies did not calculate a lag period. Faye et al. (2021) found that the relative mortality risk varied across different lags, e.g. the relative risk was below 1.0 at lag 0 days, indicating no immediate significant risk increase. However, a noticeable increase in relative risk was observed between lags 6 to 12 days, with the highest relative risk appearing at lags 8 and 9 days. The effect varied across different demographics, with significant associations among male mortality at lags 11 to 18 days and for female mortality at lags 7 to 14 days. Children aged 0 to 5 years showed significant risk at lags 8 to 14 days, and people aged 55 years or above were at a higher risk at lags 7 to 16 days. Interestingly, no significant association was observed for the age group of 6 to 54 years across different lags. This lag effect demonstrates the delayed impact of heat waves on mortality, highlighting the importance of considering varying time frames when assessing the health impacts of heat exposure in different demographic groups.

Various approaches have been used for estimating the lag period. The distributed lag nonlinear model (DLNM) is the most widely used, e.g. Nyadanu et al (2023), Bunker et al (2017), and Wiru et al (2020). The DLNM assesses the nonlinear association between heat exposure and mortality over different lag days, with a maximum lag of 25 days considered. This approach is useful for estimating heat waves' nonlinear and delayed effects on mortality. Nyadanu et al (2023) investigated the delayed effects of long-term heat stress on stillbirth rates in Ghana, using a DLNM to analyse the nonlinear exposure–response relationship and the time-structured lagged effects of heat stress. This approach is essential to understand the complex interplay between environmental factors like heat stress and adverse pregnancy outcomes. Egondi et al. (2012) employed a DLNM to understand the association between daily maximum temperature and Years of Life Lost. The study observes a J-shaped exposure–response curve, indicating a significant increase in YLL associated with cold temperatures. The study also explored the lag effect of temperature on YLL, showing that the impact of cold temperatures on YLL was observed mainly within the first five days after exposure. The study revealed no significant added impact of cold spells or heat waves on YLL beyond this lag effect. Bunker et al (2017) investigated the impact of heat exposure on non-communicable disease years of life lost (NCD-YLL) in rural Burkina Faso from 2000 to 2010. It uses a daily time series regression analysis with DLNMs. The key finding was that moderate to extreme heat exposure significantly increases premature deaths from NCDs. The most pronounced health effects were observed on the day of heat exposure, with a diminishing impact over the following four days. This lag effect demonstrates heat exposure's immediate and short-lived impacts on NCD-related mortality. Wiru et al (2020) used a DLNM to analyse the relationship between daily mean apparent temperature and all-cause mortality. The study found a nonlinear association, observing increased mortality risks at lower temperatures, especially from lag 2 to 4 days after exposure, with the highest risk occurring 3 days after exposure. This lag effect illustrates the delayed impact of temperature changes on mortality risks. The study also notes sex-specific differences in the temperature-mortality relationship.

Poisson regression is also often used to assess lag effects. Luque Fernández et al. (2009) used a Poisson autoregressive model to analyse the relationship between the weekly number of cholera cases and climatic variables. The study found a significant association between the increase in cholera cases and a rise in temperature 6 weeks prior, as well as an increase in rainfall 3 weeks before. Azongo et al (2012) used a time-series Poisson regression approach to analyse the short-term associations between mortality and mean daily temperature. They found a significant association at various lag days, indicating that temperature variations can have delayed effects on mortality.

### Distribution of studies based on urban, rural, and informal settlements.

The review identified 34 studies (34%) focusing on urban areas. 21 studies (21%) were carried out in rural areas. The remaining 45 studies (45%) assessed the association between ambient temperature or heat waves and mortality in urban and rural areas. Table 4 summarises studies that have been conducted in urban and rural areas of tropical Africa. Some studies compared urban and rural populations based on their sensitivity to extreme heat (Nunfam et al. 2021; Jankowska et al. 2012; Alexander et al. 2013). Fewer studies were carried out in rural areas compared with urban, with the rural areas of West Africa having more studies relative to other regions. Together these studies revealed the harmful influence of heat on human health, behaviour, and productivity among farmers, labourers, and mining workers in rural communities (Nunfam 2021). While populations have diverse responses and coping mechanisms to heat exposure, these are inefficient in preventing heat-related morbidity and mortality at both the household and farm levels (Frimpong et al. 2020). Urban centres are known for their heat impacts on human health due to their propensity to create heat islands. The urban heat island (UHI), whereby temperatures in urban areas are higher than in the surrounding rural regions, exacerbates the influence of heat on human health (Sheridan and Allen 2015). Urbanisation is the leading cause of urban sprawl. Urban sprawl has led to the growth of informal settlements that house low-income populations in many Tropical African cities.

**Table 4:** Classification of studies based on urban and rural areas

Sub-region	Urban and rural	Urban	Rural
West Africa	Nigeria (Olatunde 2016; Adeniyi 2009; Balogun and Daramola 2019; Omonijo 2017; Balogun and Balogun 2014; Ragatoa et al. 2018; Agada and Yakubu 2022; Eludoyin 2014, 2015; Morakinyo et al. 2016; Njoku and Daramola 2019; Tunde et al. 2013; Kiki et al. 2020); Ghana (Kwasi et al. 2014; Asamoah et al. 2018; Nunfam et al. 2019a, b; Nunfam et al. 2021; Nyadanu et al. 2023); Mali (Jankowska et al. 2012); Senegal (Thiam et al. 2017; Sarr et al. 2019); West Africa (Efeoma and Uduku 2014; Blom et al. 2022; Adeniyi and Oyekola 2017; Sylla et al. 2018)	Nigeria (Ifatimehin and Ujoh 2014, Omonijo et al. 2013, Daniel 2015, Alaigba et al. 2018; Olatunde 2016, Lorena et al. 2018, Adegebo 2022, Obe et al. 2023, Upla and Bisong 2021); Gambia (Brewster and Greenwood 1993; Jaffar et al. 1997); Senegal (Faye et al. 2021, Sy et al. 2022); Burkina Faso (Sankoh et al. 2003; Kynast-Wolf et al. 2006, 2010; Hammer et al. 2006; Diboulo et al. 2012); Ghana (Azongo et al. 2012; Dukić et al. 2012)	Nigeria (Lawoyin 2001; Oloukoi et al. 2014); Senegal (Diouf et al. 2013; Etard et al. 2004); Gambia (Bonell et al 2022, 2023; McGregor et al. 1961; Rayco-solon et al. 2004); Ghana (Nunfam et al. 2021; Frimpong et al. 2014, 2016, 2020; Nunfam et al. 2019a, b; Wiru et al. 2020; Nunfam 2021). Burkina Faso (Bunker et al 2017; Arisco et al 2023)
Central Africa	(Fotso-Nguemo et al. 2022)	Democratic Republic of Congo (Longo-Mbenza et al. 1999, Ng and Cowling 2014); Cameroon (Dapi et al. 2010; Enete et al. 2013)	
South Africa	Botswana (Alexander et al. 2013); South Africa (Maposa et al 2021; Bühler et al 2022; Kapwata et al 2021; Kunene et al 2023; Martineau et al 2022)	Zambia (Chang et al. 2004, Paz 2009, Luque Fernández et al. 2009); Zimbabwe (Chang et al. 2004; Ngwenya et al. 2018; Mutanga et al. 2018)	South Africa (Wright et al 2017a, b, Ikeda et al 2019, Kapwata et al 2018a, b, Wright et al 2017a, b, Wright et al. 2022, Thompson et al. 2022, Manyuchi et al. 2022, Mabuya and Scholes 2020, Kapwata et al. 2018a, b)
East Arica	Kenya (Scott et al. 2017; Scorgie et al. 2023); Tanzania (Ndetto and Matzarakis 2017; Gyilbag et al. 2021)	Kenya (Ye et al. 2009, Mutisya et al. 2010, Thaddaeus et al. 2012, Grace et al. 2012, Mutai 2013); Tanzania (Trærup et al. 2011; Ndetto and Matzarakis 2013; Lorena et al. 2018; Paz 2009; Reyburn et al. 2011)	Tanzania (Mrema et al. 2012); Uganda (Van de Walle et al. 2022)
Indian Ocean Island			

Informal settlements are an essential feature of tropical African cities, commonly identified as unplanned and densely-packed low-rise buildings with a high population (Yahia et al. 2018). The dwellers of informal settlements are more sensitive to the impact of extreme heat due to their low adaptive capacity, e.g. Lorena et al (2018) found an increase in non-communicable diseases in children, deteriorating mental health, and occupational hazard in adults of informal residences with low income due to extreme Temperature. The informal settlements are densely packed housing with poor building materials that lack access to public services and amenities, making their population particularly vulnerable to heat (Scott et al. 2017). The disparities in the designs of built-up expansion, vegetation and construction materials in cities can differentially affect the threat of heat-related morbidity and mortality. For instance, Egondi et al (2012) found that the extreme heat experienced in the neighbourhood of the informal settlements was more than the ambient temperature recorded in the nearest weather station by several ^o^C. A study by Scott et al (2017) employed iButtons – an inexpensive device for measuring temperature and relative humidity, to investigate heat variations in an informal settlement in Nairobi. Both Scott et al (2017) and Egondi et al (2012) recognised that poor populations were at greater risk of extreme heat than wealthier populations, highlighting the social inequalities that exist in heat exposure and adverse health outcomes. There are, however, very few empirical studies on the vulnerability of human health to heat, particularly in the informal settlements of tropical Africa (Pasquini et al. 2020). Even though climate threat is projected for the African continent (Dosio 2017), studies in Zimbabwe have shown that informal settlements and urban outdoor workers are more vulnerable to extreme heat (Ngwenya et al. 2018). To this end, very little is known about the vulnerability to heat in tropical Africa.

### Health outcomes

The primary health outcomes of the studies cited in this review are health risks associated with increased temperatures and heat stress (Joseph and Demot, 2021; Morakinyo et al. 2016; Van de Walle et al. 2022; Gratien Kiki et al. 2020; Mabuya and Scholes 2020; Sylla et al. 2018; Wright et al. 2022). For instance, Mushore et al. (2017) identified outdoor thermal discomfort in densely built-up areas, and Ndetto and Matzarakis (2013), Ndetto and Matzarakis (2017) and Sarr et al (2019) found heat stress and thermal discomfort to be the major health issues during the hot season leading to heat exhaustion, heatstroke, and overall discomfort affecting daily activities.

The review highlights the myriad of ways by which extreme heat affects human health, through different and varied health outcomes. Several studies report an increase in the incidence of diarrhoea, respiratory infections, malaria, and physiological stress associated with heatwaves, heat stress and extreme temperatures (Omonijo et al. (2011), Adeniyi and Oyekola (2017), Thandi et al. (2018), Njoku and Daramola (2019), and Adeboyejo et al. (2012)). Dukic et al. (2012) and Tunde et al. (2013) observed an increase in the prevalence of asthma, malaria, meningitis, and typhoid fever due to temperature, relative humidity, and air quality. Other studies have reported that increasing temperatures and relative humidity exacerbate heatstroke, heat stress, heat cramps, heat exhaustion, dehydration, kidney failures, acute meningitis, productivity loss, anxiety, increased risk of malaria and effects on social well-being among outdoor workers (Frimpong et al. (2014), Nunfam (2021), and Frimpong et al. (2020)).

The review also identifies several health outcomes, specifically relevant to children. Sylvia Blom et al (2022) found increased chronic and acute malnutrition in children due to extreme heat exposure. Scorgie et al (2023) found an increased risk of heat-related health issues such as heat exhaustion, dehydration, and potential impacts on foetal health. Nyadanu et al (2023) identified an increased risk of stillbirth associated with exposure to long-term heat stress. Bonell et al (2023) suggests that reducing maternal exposure to heat stress and strain will likely reduce foetal strain, potentially decreasing adverse birth outcomes.

Some studies have shown how extreme heat disproportionally affects the elderly and female population, e.g. Faye et al (2021) found that heat waves lasting three or more consecutive days increase the risk of death, with the elderly over 55 years and females being more affected.

### Priorities for reducing the health impacts from extreme heat

There is a need for a more detailed analysis of cause-specific mortality to understand better and address regional seasonal mortality patterns in tropical Africa (Ndetto and Matzarakis 2013; Ndetto and Matzarakis 2017; Lawoyin 2001; Kynast-Wolf et al. 2005; Mutisya et al. 2010; Diboulo et al. 2012; Azongo et al. 2012; Mrema et al. 2012; Scott et al. 2017; Wiru et al. 2020).

Several studies emphasise the importance of integrating tree planting and urban greening in building and urban design and materials to enhance thermal comfort and ventilation to enhance thermal comfort and reduce health risks associated with extreme temperatures, particularly in regions where heat stress has a major impact on human health and productivity (Omonijo et al. (2013), Morakinyo et al. (2014), Njoku and Daramola (2019), Mushore et al. (2017), Mabuya and Scholes (2020) and Van de Walle et al. (2022)). Wright et al (2022) emphasise the need to develop climate-proof housing and improve access to essential services to support resilient coping mechanisms, particularly in rural areas, during heatwaves. Ndetto and Matzarakis (2013) prioritise adapting urban planning and architectural design to mitigate heat stress, including optimising street orientation, and building heights to enhance thermal comfort in urban areas.

Moreover, several studies have underscored the importance of implementing effective adaptation measures (Egondi et al. (2012), Dukic et al. (2012), Adeboyejo et al. (2012) and, Adeniyi and Oyekola (2017), Sarr et al. (2019)). These include enhancing public awareness, improving public health infrastructure, developing health action plans, enhancing disease surveillance and response systems, increasing community awareness, preparedness and education on health risks associated with climate change, and targeting children.

This review also finds that further research is needed to quantify better the impact of warming on socioeconomic activities and health, to inform more targeted and efficient adaptation strategies, which is crucial for mitigating the adverse effects of heatwaves and extreme temperatures on human health. Sylvia Blom et al (2022) suggested implementing healthcare and nutrition program strategies to reduce the impact of rising temperatures on child nutrition. Adeniyi and Oyekola's (2017) argue that improving regional climate modelling is a priority for better prediction of heat waves. Tunde et al. (2013) recommend public awareness and education about climate variability and its effects on health through broadcasting weather reports and educating people on the impacts of anthropogenic activities on the climate.

Additionally, practical measures using mosquito nets, clearing stagnant water, and avoiding residing near riverbeds are suggested to reduce the risk outcomes from future heatwaves. Future priorities for minimising these risks involve implementing heat stress management strategies, enhancing workplace heat stress policies, and improving awareness and training about heat-related health risks among outdoor workers (Ngwenya et al. 2018; Nunfam 2021). Future priorities for reducing these health risks include developing effective heat wave early warning systems and public health strategies tailored to the needs of the most vulnerable groups, such as the elderly, children, and female population, to enhance preparedness and response to heat waves and mitigating their impact on human health (Faye et al. 2021).

Adapting to extreme heat should take account of evidence from this review that extreme heat can affect female populations more (Faye et al. 2021). Scorgie et al (2023) emphasise the importance of developing culturally appropriate adaptation strategies to reduce heat risks for pregnant women. These strategies should consider local gender dynamics to empower women, enhance their autonomy, and improve community support during hot seasons. Nyadanu et al (2023) emphasise the need for public health and climate governance strategies to reduce maternal exposure to heat stress, particularly in rural areas, to lower the risk of stillbirth. These strategies may include developing heat stress warning systems, improving maternal healthcare services, and enhancing awareness and education about the risks of heat exposure during pregnancy. Bonell et al (2023) prioritises further research to explore the association between heat stress and pregnancy outcomes in various settings and populations, aiming to develop effective interventions.

### Opportunities for improving study methodologies

The majority of studies cited in the review obtained the climate data from traditional weather stations (Trærup et al. 2011; Reyburn et al. 2011; Adeniyi 2009; Eludoyin 2014; Dukic et al. 2012), which means the estimates of climate are not necessarily identical to the conditions experienced by the population. This is because people experience thermal discomfort indoors as well as outdoors, in distinct locations which may be a significant distance from the outdoor weather station. Our review has highlighted the importance of understanding thermal stress in informal settlements, yet temperatures are rarely monitored in these settings. Moreover, many urban areas of tropical Africa lack dense in-situ monitoring networks that can provide air temperature data at high spatial resolution.

Advances in technology offer an opportunity to address some of the methodological gaps discussed above. It is possible to measure climatic conditions more closely to the populations being affected, even at the individual person level. For example, few studies have used wearable devices such as iButtons that collect data on air temperature, humidity, and UV radiation (Scott et al. 2017; Mabuya and Scholes 2020; Van de Walle et al. 2022) or EasyLog-USB and Lascar USB temperature and humidity sensors (Kwasi et al. 2014; Balogun and Balogun 2014; Frimpong et al. 2016; Kiki et al. 2020). Moreover, recent technological advancements have led to the development of intelligent sensors like microneedles, skin patches, tattoos, and stretchable electronics. These devices can monitor various physiological parameters, including sweat rate, sodium levels in sweat, skin temperature, and heart rate (Paulo Silva Cunha 2018) and facilitate the creation of Internet of Things (IoT) networks to measure environmental conditions (Chapman 2015).

Remotely sensed data from satellite observations provide greater spatial coverage of land surface temperatures than what can be achieved with traditional weather station data. However, only 2% of the articles cited in this review used freely accessible satellite thermal imagery to map land surface temperature (Ifatimehin and Ujoh 2014; Scott et al. 2017; Mushore et al. 2017; and Van de Walle et al. 2022). Thermal bands of satellite imagery such as Landsat, MODIS, and Sentinels, provide datasets with spatial resolution from 10 m to 1 km, enabling potentially high resolution thermal mapping in urban areas. Although Landsat 5–9 imagery has good spatial resolution (100 m), the image is acquired at 10.00 am, which is unsuitable for heat-human health studies because maximum temperatures occur later in the day and minimum temperatures earlier. Although satellites can provide high resolution temperature data, they provide estimates of land surface temperature, which is not the same as air temperature, and a conversion is necessary (Anderson et al. 2021; Wang et al. 2022; Khan et al. 2022).

## Conclusions

The evidence gathered from 100 articles in this review revealed that dehydration, discomfort, and heat-related morbidity and death increased during high temperatures or heat waves. The harmful effects of extreme heat on human health in tropical Africa include declining mental health in adults of low-income residents (Lorena et al. 2018), an increase in miscarriage risk with each degree of temperature rise (Asamoah et al. 2018), and effects on the safety and well-being, psychological behaviour, productivity, and social comfort of outdoor workers who spend long hours performing manual labour (Nunfam et al. 2019a, b; Nunfam 2021). The findings of this study are consistent with previous findings that higher temperatures increase the incidence of morbidity. According to Liu et al (2021b), renal disease rose by 10% for every 1°C increase. Heat and mental health research evaluations show that morbidity rose by 0.9% to 22% for every 1°C increase (Liu et al. 2021a). According to Phung et al (2016), the risk of cardiovascular hospitalisation increased by 0.7%. According to Fatima et al (2021), occupational illnesses and injuries increase by 1% for every 1°C increase in temperature. Regarding morbidity or mortality, Faurie et al (2022) reported over 100% increases in case numbers. Given that more than 90% of urban population growth is anticipated in Asia and Africa (UN-Habitat 2014), urbanisation and increases in the artificial impervious surface are anticipated to impact the thermal environment due to the destruction of vegetation cover and the expansion of informal settlements. Almost 55% of Sub-Saharan Africa, according to UN-Habitat (2014), resides in informal settlements, which are more vulnerable to heat-related morbidity and mortality due to their dense population and poor living conditions. Heat-related health impacts are of concern in tropical Africa, which is already facing substantial heat stress due to the climate and environmental change exacerbated by anthropogenic activities and increasing greenhouse gas levels.

The impact of extreme heat on human health in tropical Africa is worsened by the population's relative poor socioeconomic and demographic status and the environmental quality. Green spaces are an essential contributor to human well-being. Studies have found that people dwelling in areas with less vegetation cover are more vulnerable to heat-related morbidity and mortality (Schinasi et al. 2018). Informal settlements are characterised by poor physical infrastructure and little vegetation cover, which influences the UHI effect and increases night-time temperatures. Nighttime cooling is essential for people to get a good night's sleep and recuperate from the day's heat. There is a strong association between amplified night-time heat and inadequate sleep; the consequence is more prevalent among the lower-income and ageing population (Obradovich et al. 2017). Most of the studies cited in this review reported increased heat-related morbidity and mortality during the dry/hot season and the heat/rainy season relative to the dry/cold season. For example, the prevalence of infectious diseases, such as malaria and diarrhoea, increased mortality in coastal towns of tropical Africa during the hot/wet season (Greenwood 1993; Ifatimehin and Ujoh 2014). The common reasons for excess mortality in these seasons are extreme heat and hygienic environments. The increased rain usually overstretches the sewage and drainage systems, leading to stagnant water and a wet environment.

Moreover, stagnant water and a damp environment offer numerous disease agents decent breeding and surviving grounds. In addition, the heat/rainy season, characterised by planting and growing crops, often coincides with the time of least food supply and poor nutritional status of the population (Rayco-solon et al. 2004). The dry/hot season is the transition period between Harmattan and the heat/rainy season in tropical African cities that border the Sahara Desert. The dry/hot season exhibits excess mortality due to extreme heat, increasing the time spent outdoors to try and cool down. The poor population that cannot afford air conditioning spends more time outside, making them vulnerable to disease pathogens (Pasquini et al. 2020). Moreover, their dwelling is usually overcrowded and poorly ventilated, leading to indoor air pollution, a significant cause of mortality peaks in informal settlements. There is a further increased risk of airborne disease and meningitis due to the Harmattan dust from the Sahara Desert during the dry/hot season. A common observation in most identified studies in tropical Africa was the age dependency of morbidity seasonality. Older people are at a higher risk of dying during the hot/dry season (Daniel 2015; Scott et al. 2017), while children below 9 years are most vulnerable to death in the heat/rainy season (Kynast-Wolf et al. 2006). Human sensitivity and ability to adapt to extreme heat's effects depend on the population's demographic and socioeconomic status. Generally, there is a link between the human dwelling environment, the socioeconomic characteristics, and the adverse effects of extreme heat.

### Supplementary Information

Below is the link to the electronic supplementary material.

Supplementary file1 (DOCX 37 KB)
