# Supplementary material for: The effects of extreme heat on human health in tropical Africa
Source: Int J Biometeorol. 2024 Mar 25;68(6):1015–33. doi: 10.1007/s00484-024-02650-4 (PMC11108931; doi:10.1007/s00484-024-02650-4)
Supplement: Supplementary file 1 — Supplementary file1 (DOCX 37 KB) [file 484_2024_2650_MOESM1_ESM.docx]

Table (Online resources 1) Summary of studies on the effects of extreme heat on human health in tropical Africa

| Reference | Location/Country | The type of data used and data analysis techniques | Summary of findings | Lag period |
| --- | --- | --- | --- | --- |
| Wright et al (2017) | Mopani, Limpopo Province, South Africa | Temperature and relative humidity recorded at 30-min intervals inside the clinics' main indoor patient waiting areas. Pearson correlation coefficients and linear regression were used to examine the relationship between indoor and outdoor temperatures. | The study found that indoor temperatures in waiting rooms were significantly warmer than outdoor temperatures, especially during summer months, with mean ATs indicating that patients and healthcare workers may experience discomfort and health risks due to elevated temperatures. | Not mentioned |
| Maposa et al (2021) | Limpopo province, South Africa | Monthly maximum temperature. Bivariate conditional extremes modelling with a time-varying threshold approach, employing penalised cubic smoothing spline for nonlinear detrending of temperature data and maximum likelihood estimation (MLE) for parameter estimation. | Thabazimbi showed a strong positive extremal dependence on high-temperature values at Mara, while Polokwane exhibited strong negative extremal dependence on high-temperature values at Messina. | Not mentioned |
| Kapwata et al (2018) | Greater Giyani, Limpopo Province, South Africa | Hourly indoor temperature and relative humidity and Outdoor temperature, humidity, and wind speed. Apparent Temperature (AT) calculations and multiple linear regression | Seasonal trends in indoor and outdoor temperatures with high indoor AT pose potential health risks. Future predictions indicate an increase in indoor AT, exacerbating health risks in vulnerable rural communities. | Not mentioned |
| Bühler et al (2022) | Mopani, Limpopo Province, South Africa | Hospital admissions for cardiovascular diseases (CVD), temperature and relative humidity. Distributed lag non-linear model (DLNM) with a negative binomial regression over a 21-day lag period. | Warm and cold apparent temperatures (Tapp) are associated with an increase in hospital admissions for CVD, with cold Tapp (6–25°C) and warm Tapp (27–32°C) showing significant effects. Cumulative effects over 21 days indicated a larger fraction of CVD admissions attributable to non-optimal temperatures. | 21 days |
| Kapwata et al (2021) | Limpopo Province, South Africa | Temperature, rainfall, air pollution, hospital admissions for diarrhoeal disease, malaria, pneumonia, and asthma. Wavelet transform cross-correlation analysis. | Significant associations between changes in air quality and pneumonia cases and between climatic conditions (heat and rainfall) and malaria | 10-15 days |
| Kunene et al (2023) | Mopani, Limpopo Province, South Africa | Ambient temperature and daily hospital admissions for diarrhoea. Linear and threshold regression analyses | The study found an increase in diarrhoea admissions with each 1°C rise in temperature for all ages, particularly in individuals over five years old. | Not mentioned |
| Ikeda et al (2019) | Mopani, Limpopo Province, South Africa | Temperature, precipitation and diarrhoea case counts. Contour analysis | Significant patterns were found between climate conditions and diarrhoea case counts, especially in children under five years. Both arid and wet conditions were associated with higher case counts. | Up to 8 weeks |
| Kapwata et al (2018) | Giyani, Limpopo Province, South Africa | Particulate matter (PM4) concentrations and temperature. Descriptive statistics were used to explore seasonal and diurnal variations. Pearson’s correlation coefficient (r) was employed to assess the relationship between PM4 concentration and indoor and outdoor temperature. | The findings highlight significant seasonal variations in PM4 levels, with indoor temperatures positively correlating with PM4 concentrations during warmer months (r) of 0.22: p < 0.001 and spring (r) of 0.24: p < 0.001. | Not specified |
| Wright et al (2017) | Limpopo Province, South Africa | Blood collection and sun exposure diaries. Chi-squared test, t-test, multiple linear regression. | Sun protection intervention did not significantly alter the findings, indicating the complexity of factors influencing vaccine effectiveness. |  |
| Bonell et al (2022) | West Kiang region, The Gambia | Environmental temperature, humidity, maternal heart rate, skin temperature, fetal heart rate, and umbilical artery resistance index. Linear regression for fetal heart rate and logistic regression for fetal strain. | Exposure to extreme heat was associated with fetal strain. Decreasing maternal exposure to heat stress and strain is likely to reduce fetal strain, potentially reducing adverse birth outcomes. | Not specified |
| Martineau et al (2022) | Limpopo, South Africa | Malaria case and climate data from the NCEP/NCAR reanalysis, CPC Merged Analysis of Precipitation (CMAP), and NOAA OISST datasets. Machine learning classification models, lag-regression analysis and nested cross-validation for model evaluation. | The study found that sea surface temperature variability in the Pacific and Indian Oceans can predict malaria outbreaks in Limpopo up to 9 months in advance with significant accuracy, demonstrating the potential for climate information in early planning interventions against malaria outbreaks. | Not specified |
| Fotso-Nguemo et al (2022) | Central Africa | The discomfort index calculated using Thom's formula and the heat index calculated using Rothfusz's equation. Estimations produced within the framework of the Coordinated Output for Regional Evaluations project that are based on an ensemble-mean of 8 RCM simulations. | Days with potentially dangerous heat-related risks have emerged because of persistently high temperatures, when nearly everyone experiences heat exhaustion. | 28 days for all exposure |
| Morakinyo et al (2014) | Akure, Nigeria | Indoor and outdoor air temperature and relative humidity. The study utilised the Temperature-Humidity Index (THI) and the Relative Strain Index (RSI) for analysing thermal comfort. | The study found that tree shading improves indoor and outdoor thermal comfort, demonstrating the role of vegetation in microclimatic control. | Not mentioned |
| Tunde et al (2013) | Ilorin, Nigeria. | Rainfall, relative humidity, temperature, sunshine, and health data (incidence of asthma, malaria, and typhoid). Correlation, regression, and Analysis of Variance (ANOVA). | The study found significant relationship between climatic conditions and the prevalence of asthma (*R*20.49), typhoid (*R*20.88), and malaria (*R*20.79). | Not mentioned |
| Van de Walle et al (2022) | Kampala, Uganda | Temperature, relative humidity, and NDVI. Multiple linear regression with stepwise backward elimination. | The study found that areas with low vegetation in Kampala experience higher heat stress. NDVI was the most influential predictor of intra-urban variations in daily mean heat stress. | Not specified. |
| Mushore et al (2017) | Harare, Zimbabwe | LST, humidity and air temperature. Linear regression and discomfort index analysis | The study found high discomfort in densely built-up areas, while areas with more vegetation experienced less discomfort. | Not specified. |
| Blom et al (2022) | West Africa | Demographic and Health Surveys data and temperature. Econometric analysis linking demographic data with temperature exposure. | Extreme heat exposure increases the prevalence of chronic and acute malnutrition in children. A 2°C rise in temperature was predicted to increase the prevalence of stunting, reversing progress made in improving child nutrition during the study period. |  |
| Njoku and Daramola (2019) | Southwestern region of Nigeria | Air temperature, relative humidity, wind speed, and solar radiation. Analysis of THI, PET, seasonal and decadal variation analysis. | The study found variations in thermal comfort in the region, with seasons affecting outdoor thermal comfort levels. | Not specified. |
| Mabuya and Scholes (2020) | Gauteng and Mpumalanga, South Africa | Ambient temperature. ANOVA, linear regression, and Pearson’s correlation. | Variations in indoor temperatures among different low-cost housing types, influenced by construction materials and design, with implications for thermal comfort and energy use. | Not specified |
| Wright et al (2022) | Limpopo Province, South Africa | Temperature, questionnaire responses on thermal comfort and coping mechanisms. Descriptive analysis and logistic regression | Participants perceived their dwellings as too hot. Common coping mechanisms included sitting in shaded outdoor areas and opening windows. | Not specified |
| Adeboyejo et al (2012) | Limpopo Province, South Africa | Temperature, rainfall, and hospital records for Diarrhea, respiratory infections, and malaria in children. Regression analysis, chi-square test and Pearson correlation. | Diarrhea, respiratory infections, and malaria are influenced by temperature, rainfall. | Not specified |
| Kiki et al (2020) | Cotonou, Benin. | Temperature, relative humidity, air velocity and survey data from office building occupants. Fanger’s PMV model, adaptive models of Lopez-Perez and Indraganti, PMVnew, and aPMV for thermal comfort evaluation. | The study found that Fanger's PMV model underestimates the adaptability of occupants to high temperatures. Adaptive models provided a more accurate assessment of thermal comfort in the studied office building. | Not specified. |
| Ibu and Bisong (2021) | Calabar, Nigeria. | Temperature and socio-cultural data (questionnaire responses). Chi-square and ANOVA. | The study indicates variations in bioclimatic discomfort among different age groups, with implications for urban planning and public health | Not specified |
| Alexander et al (2021) | Botswana | Temperature, rainfall, vapor pressure and health data (diarrheal disease reports). Autoregressive analysis of covariance (ANCOVA), stepwise variable selection. | Diarrheal disease shows a significant correlation with temperature and rainfall. | One month |
| Manyuchi wt al (2022) | Agincourt sub-district, South Africa. | Qualitative data collected through interviews and structured observations. Thematic analysis with qualitative methods. | The study found increased perception of heat affecting labour productivity and health. | Not mentioned. |
| Agada et al (2022) | Yobe State, Nigeria. | Temperature and relative humidity. Time series analysis and linear regression. | The study found an increase in extreme heat from 2009-2020, with heatwaves significantly impacting human life. | Not mentioned |
| Sarr et al (2019) | Senegal | Temperature projections from regional climate models. Statistical analysis of temperature extremes and heat indices. | Projected increases in temperature extremes, with impact on human health due to rising heat waves and heat indices. | Not mentioned |
| Ngwenya et al (2017) | Bulawayo, Zimbabwe | FGD (with health practitioners and policymakers) and temperature. Grounded theory | The study revealed an increase in temperatures, lack of heat stress prevention policies, and a need for improved heat management strategies and education for outdoor workers, to mitigate heat-related health risks. | Not mentioned |
| Dukic et al (2012) | Navrongo, Ghana | Dust status, sunshine hours, temperature, humidity, rain, wind speed), CO_2_ emission estimates and monthly counts of meningitis and pneumonia. Generalized Additive Models (GAM) and Generalized Linear Models (GLM) | Temperature, relative humidity, and CO_2_ emissions due to fires were found to be associated with meningitis incidence. High temperatures and low humidity increased meningitis risks, while increased humidity had a protective effect. | The previous month and from two months ago |
| Eludoyin (2014) | Nigeria | Day-time relative humidity, temperature, and questionnaires. Moving averages technique and descriptive mapping. | The study found spatiotemporal variation in thermal stress with both heat and cold stress. | Not specified |
| Eludoyin (2015) | Tertiary institutions in Nigeria | Air temperature, relative humidity, and questionnaire survey. Descriptive statistics, ANOVA, regression analyses, GIS mapping. | Physiologic comfort in Nigeria shows spatiotemporal variation in the perception of thermal comfort and coping strategies influenced by geographic location, season, and individual socioeconomic status. | Not specified |
| Ndetto and Matzarakis (2017) | Dar es Salaam, Tanzania. | Air temperature, relative humidity, and thermal comfort questionnaire. Binning method for mean thermal sensation votes (MTSV), and regression analysis. | The study found variations in human thermal perception in different environments within the city, influenced by solar radiation, wind speed, and relative humidity. | Not specified |
| Adeniyi and Oyekola (2017) | West Africa | Daily minimum and maximum temperature, Reanalysis mean daily sea level pressure, winds, and geopotential height. heat and cold wave calculation using excess heat factor (EHF) and excess cold factor (ECF), statistical tests for model validation. | The study identifies patterns and dynamics of heat and cold waves in West Africa, showing variations in frequency and amplitude linked to regional climatic factors. The study validates the effectiveness of regional climate models in simulating temperature extremes. | Not specified. |
| Scorgie et al (2023) | Kenya, Kilifi | In-depth interviews, FGD, and key informant interviews with pregnant women, spouses, and health workers. Grounded Theory and thematic analysis. | Extreme heat impacts pregnant women’s physical and mental well-being, with symptoms such as heat exhaustion, dehydration, and irritability. | 1-14 days |
| Arisco et al (2023) | Burkina Faso, Nouna region | Cause-of-death data from the Health and Demographic Surveillance System, temperature, and precipitation. Distributed-lag zero-inflated Poisson models | High temperatures and low precipitation increased the risk of death from climate-sensitive diseases, particularly malaria. | 7-14 days |
| Sylla et al (2021) | West Africa | Model simulations. Heat stress analysis using heat indices | Under 1.5°C and 2°C global warming scenarios, extreme caution heat stress increases, affecting human comfort and health in the Sahel and Gulf of Guinea. | Not specified |
| Nyadanu et al (2023) | Ghana | Stillbirth Universal Thermal Climate Index (UTCI) data. Distributed lag nonlinear models and conditional quasi-Poisson regression | Moderate heat stress increases the risk of stillbirth, particularly in rural areas with lower population density and GDP. The risk was higher during the dry winter season compared to the rainy summer season | 9-10 months |
| Oluwafemi et al (2023) | Nigeria, Lagos | Humidex, relative wealth index, demographics of the population and proportion of vegetation (PV). Optimised Hot Spot Analysis (OHSA), Moran’s I test, and Geospatial analysis. | Heat risk in densely populated and industrialised urbanised areas. | Not specified |
| Adegebo (2020) | Ibadan, Nigeria | Minimum and maximum temperature, relative humidity, and survey on the perceptions of ambient thermal conditions, comfort levels, health effects, coping strategies, and suggestions for improving city thermal conditions. Multivariate analysis. | Increased urban temperatures and perception of thermal discomfort; major health effects include dehydration, heat exhaustion, sleep disturbances; coping strategies affected by socio-economic factors. | 9-10 months |
| Gyilbag et al (2022) | Tanzania | Temperature and climate simulations from CORDEX-Africa RCMs. Time series analysis using Heatwave Magnitude Index daily (HWMId). | Heatwaves increased marginally in Tanzania from 1983-2016. The intensity and duration of heatwaves are predicted to rise in the 21^st^ Century, with higher-level heatwaves becoming more frequent and prolonged by 2041. | One month |
| Bunker et al (2017) | Nouna, Burkina Faso | Temperature and Cause of death established by verbal autopsy. Time series quasi-Poisson regression using a distributed lag non-linear model (DLNM). | Moderate and extreme heat exposure increase the years of life lost (YLL) from non-communicable diseases (NCD), especially in men. The strongest health effects manifested on the day of heat exposure. | 14-28 days |
| Meshack et al (2014) | Enugu, Nigeria | Pilot research data from five office buildings. Use of ASHRAE Thermal Comfort Standard | ASHRAE adaptive model is more suitable for assessing thermal comfort in tropical West African office buildings. | 14-28 days |
| Bonell et al (2023) | West Kiang, The Gambia | Observational study of pregnant women involved in farming, measuring heat stress, maternal heat strain, and fatal response. Multivariable repeated measure models (linear and logistic regression) | Extreme heat exposure was associated with fatal strain. Decreasing maternal heat stress and strain is likely to reduce fatal strain, suggesting a potential to lower adverse birth outcomes. | 14 -21days |
|  |  |  |  |  |
| Faye et al (2021) | Banda Fassi, Senegal | Daily temperatures, dew point temperature, wind speed, rainfall, mortality count and Demographic Surveillance System. Poisson GAM and Distributed Lag Non-Linear Model (DLNM) | Heat waves increased mortality risk, especially for females and older people | 25 days |
| Diouf et al (2013) | Ferlo, Senegal | Rainfall, temperature Clinical data. Liverpool Malaria Model, Spectral and Filtering analysis. | Temperature and rainfall influence the epidemiology of malaria through their development rates, survival of pathogens, and changes in land surface characteristics | Not specified |
| Étard et al (2004) | Senegal | Monthly mortality rate, Verbal autopsy, Post-mortem interview, and questionnaire. Independent review by two physicians to assign the principal reason for demise. Discordant diagnoses were discussed by the panel of clinicians and a consensus cause was reached. | Diarrhoeal, malaria, and acute respiratory infections called for 30% and 70% of the death earlier than 10 years of age in the warming time. | Not specified |
| Nunfam et al (2021) | Western Region of Ghana | Descriptive cross-sectional survey. Confirmatory factor and invariance analysis | Extreme heat has a major effect on well-being and behavioural efficiency. | Not specified |
| Nunfam (2021) | 5 mining locations in the Western Area of Ghana. Ghana | Surveys and mixed methods approach with quantitative (survey) and qualitative (FGD). Thematic analysis and descriptive statistics involving frequency, percent, and inferential statistical | Workers are sensitive to extreme heat due to their occupational practices, which affects their well-being and safety, psychological behaviour, efficiency, and social comfort. | Not specified |
| Wiru et al (2020) | Ghana, Kintampo area | Temperature, relative humidity, and daily counts of all-cause mortality. over dispersed Poisson regression, Distributed Lag Nonlinear Models. | Nonlinear association between mean daily apparent temperature and all-cause mortality; increased risk of death at lower apparent temperatures, with males sensitive to temperature extremes, females more vulnerable to low-temperature mortality. | 2-4 day |
| Frimpong et al (2020) | Bawku East, Ghana | Household survey and FGD. Descriptive and inferential statistics (chi-square). | Malaria and heat cramps are repeated illnesses among farmers due to heat stress. | Not specified |
| Nunfam et al (2019a) | Ghana | Questionnaires and interview. Descriptive statistics, chi-square, and Fisher's exact tests | A significant association between educational attainment and adaptation strategies to occupational heat stress (*p < 0.05*). | Not specified |
| Nunfam et al (2019b) | 5 mining spots in Western Ghana | Questionnaires and FGD. Descriptive and inferential statistics. E.g. frequency and percent, chi-square, fisher exact test. | Temperature-connected illnesses varied with the type of mining activity (*p < 0.001*). | Not specified |
| **Frimpong et al (2016)** | Bawku, Ghana | Household survey and FGD. Correlation test. | 94 % of farmers' health are affected by heat with older farmers directly vulnerable to heat-related illnesses | Not specified |
| Frimpong et al (2014) | Manga, Garu, Binduri and Pusiga, Ghana | *Tmin*, *Tmax*, and relative humidity. Regression and Durbin-Watson statistical test. | 1^o^C increase in *Tmax* with effects on the health of outdoor farmers who spend many hours doing manual work. | Not specified |
| Kwasi et al (2014) | Pusiga, Binduri, and Manga, Ghana | WBGT. Correlation test | 93.5% of respondents expressed concern about the effects of heat on their health, 49% expressed that the increase in heat has affected their income. | Not specified |
| Asamoah et al (2018) | Upper East, West, Accra, and Central Ghana | Temperature, humidity, heat radiation, wind speed, maternal morbidity, and mortality, survey, verbal autopsy, and questionnaire. Logistic regression and the Liljegren method. | 42% rise in the odds of suffering a miscarriage with each degree rise in WBGT (crude OR 1.42 95% CI 1.00–2.03). | Not mentioned |
| Azongo et al (2012) | Kassena-Nankana,  Ghana | Temperature, precipitation, and mortality data. Time series Poisson regression, sensitivity analysis. | Strong association of mean daily temperature and precipitation with mortality; higher mortality risks associated with temperature extremes and increased precipitation; distinct effects observed across different age and gender groups | - 1. day   2-6 days  7-13 days  14-27days |
| Trærup et al (2011) | Tanzania | Temperature, Cholera cases, and mortality data. Time series, regression, and Correlation model. | A 1.0°C rise in *Tmax* rises the relative risk for cholera cases by 29% (β = 0.256, p = 0.02; IRR = 1.29) and 15% (β = 0.141, *p* = 0027; IRR = 1.15 | Not specified |
| Lorena et al (2018) | Dar es Salaam, Tanzania | Temperature and health data. Simulations | Heat-related mortality from non-communicable diseases in children. Declining mental and occupational health in adults of low-income residents. | Not mentioned |
| Mrema et al (2012) | Rufiji, Tanzania | Rainfall, temperature, mortality data from health and vital events registration system. Time-series and Poisson regression models. | A strong association between *Tmax* and death in all ages. Age group < 4 = (*RR*0.93, 95% CI0.8940.974), age group > 60 (*RR*0.946, 95% CI 0.91 0.98). | 4 months |
| Ndetto and Matzarakis (2013) | Tanzania, Dar es Salaam | Air temperature, relative humidity, wind speed, and global radiation. RayMan model. | The study found that street orientation and building height influence thermal comfort at pedestrian level, with specific orientations and heights providing more favourable thermal conditions. | Not specified |
| Reyburn et al (2011) | Unguja and Zanzibar, Tanzania | Temperature, rainfall, relative humidity, and cholera surveillance record. Time series, correlation, and autoregressive integrated moving averages model. | A 1°C rise in temperature at a 4-month lag occasioned a 2-fold rise in cholera. Heat and precipitation interface conceded a positive relationship (*P* < 0.04) with cholera at a 1-month lag. | 4 months |
| Adeniyi (2009) | Ibadan, Nigeria | Temperature Humidity Index (THI) and Wind Chill Index (WCI). Thom (1959)’s discomfort index, Fourier analysis, modified Civitan's (1995) method of harmonic analysis. | Uncomfortable conditions in the afternoon of March and June due to high temperature and humidity Index values. | Not specified |
| Lawoyin (2001) | Lagun, Nigeria | Infant birth and death information from Voluntary Health Workers, and Traditional Birth Attendants. Fisher's exact test and relative risk (RRs) | More neonatal deaths occurred during the warm and rainy seasons than in the dry season (*p*=0.02) predominantly among females (*p*=0.01). | Not specified |
| Daniel (2015) | Akure, Nigeria | Temperature, Precipitation, and incidence of heat rashes. Line graph and correlation analysis | A positive relationship between *Tmax*, rainfall, and heat-rash during Jan-Feb (0.95), Mar (0.67), Apr (0.65), Jul (0.67), Aug (0.87), Sept (0.74), and Dec (0.86). | Not specified |
| Ragatoa et al (2018) | Five Climatic zones of Nigeria | Temperature and Precipitation. Spatio-temporal trend analysis, Annual Aggregated time series | The hottest days of heatwaves vary from 26 to 45.0°C with impacts on human health. | Not specified |
| Olatunde (2016) | Lokoja, Nigeria | Temperature and relative Humidity. Thom’s Discomfort Index and the Humidex Calculator | Thermal discomfort varied from under 50% to over 50% between “noticeable” discomfort and “evident” discomfort. | Not specified |
| Alaigba et al (2018) | Akure, Nigeria | Temperature, relative humidity, wind speed. Rayman model | *Tmax* of 35.0°C – 55.0°C has led to thermal discomforts and heat stress with heart-related diseases such as fatigue, sunstroke, muscle cramps, heat stroke, and heat exhaustion | Not specified |
| Ifatimehin and Ujoh (2014) | Lokoja, Nigeria | Land use, LST, and NDVI. Hospital records for malaria infectious data. correlation and buffer analysis | The temporal increase in LST for sand bars, vacant land, and built-up area results in a rising incidence of malaria during the rainy season. | Not specified |
| Omonijo et al (2013) | Ondo, Nigeria | Temperature, relative humidity, radiation, and wind velocity. Rayman model | Moderate heat stress 57.5%, strong heat stress 32.6%, and minor heat stress 9.8%; no thermal stress 0.02%. | 1- 21 days |
| Balogun and Balogun (2014) | Akure, Nigeria | Thermohydrometric index (THI), discomfort index (DI), and relative strain index (RSI) defined by Temperature and humidity. Descriptive statistics with a one-way analysis of variance, Hemispherical images calculations using *Chapman et al*. (2001) method. | Urban core signifying major heat stress and health risk (*p < 0.05*). | Not specified |
| Oloukoi et al (2014) | Iseyin, Okeho and Shaki, Oke-Ogun, Nigeria | Temperature, rainfall, and cases of diseases from Correlational study Household survey, FGD, Key informant interviews. Pearson's R correlation test | The occurrence of Diarrhea, measles, and malaria was in the dry season. Increase cases of flu at the onset of Harmattan and the monsoon season. | Not specified |
| Omonijo (2017) | Ibadan, Nigeria | Air temperature, relative humidity, wind speed, and solar radiation. Rayman model | 43.5% moderate heat stress, 30% strong heat stress, 18.1% slight heat stress, 6.5% is under no thermal stress and extreme heat stress accounted for 1.3% and 0.6%. | Not specified |
| Balogun and Daramola (2019) | Akure, Nigeria | Air temperature, relative humidity, and dew point temperature. Thermal comfort index developed by Thom (1959). | March is the most thermally stressful of the year | Not specified |
| Ye et al (2009) | Nairobi, Kenya | Temperature, relative humidity, and rainfall. Logistic regression | Pneumonia was the top reason for mortality causing 25.7% of deaths over < 5 years between April and June | Not specified |
| Scott et al (2017) | Kibera, Mathare and Mukuru, Kenya | LST, relative humidity, and heat index. Time-series and single-channel algorithm. | Death at age 0-4 and <50 yrs. rise by 1% for every 1.0˚C rise in mean daily temperature above 20.0˚C | - 1. day   2-6 days |
| Grace et al (2012) | Kenya | Rainfall, temperature, maternal data, educational level, household water source, floor material, and livelihood zones/strategy. Cluster analysis multi-level linear regression | No influence of heat on inhibiting disparity (β = −0.03, p N 0.1 for average temperature, and β = 0.09, p N 0.1 for heat inconsistency) | Not specified |
| Egondi et al (2012) | Nairobi, Kenya | Temperature, rainfall, and mortality. Time series distributed lag approach model | 1.0°C rise in temperature over the 75^th^ percentile was related to death in under 5 years from non-communicable disease. 1.0°C reduction in temperature below the 25th percentile (threshold = 17.9 °C) was related to a 3%, 9%, and 13% rise in all-cause death in <50 years at lag 0–1 day | 14- 21 days |
| Mutisya et al (2010) | Nairobi, Kenya | Quarterly relative risk. Poison regression | Mortality is highest in rainy/hot season among new-borns and age < 5 years (RR = 1.6, CI:1.3–2.2 and RR = 1.5, CI: 1.1–2.0) | Not specified |
| Alexander et al (2013) | Botswana | Rainfall, temperature, vapour pressure, and Diarrhea occurrence. Time series and Autoregressive analysis | Increase in Diarrhea occurrence in the dry season (proportion deviation from the yearly seasonal mean [Pmy] = 0.07) but in the wet season (Pmy = −0.01) | 1 month |
| Kynast-Wolf et al (2010) | Nouna, Burkina Faso, | Temperature, Cardiovascular and all-cause death from Verbal autopsy. Time series, Poisson regression | High mortality for all-cause death, > 65 yrs was highly significant (β = 0.07, p b 0.00). Between 40 and 64 years (β = 0.03, p = 0.09) For CVD death, the influence were not statistically significant (β = 0.01, p = 0.3 and β = 0.06, p = 0.2 for 40–64 and <65 years. | 14 -21 days |
| Diboulo et al (2012) | Nouna, Burkina Faso, | Temperature, rainfall, and mortality data from Verbal Autopsy. Time series Poisson regression models | A 1.0°C rise in temperature at lag 0–1 was related to a 2.6% increase in the danger of death in all age groups. Increase in danger of 3.7% (95% CI: 0.3, 7.3). For lags 2–6 and 7–13 among under 5 years. The old <60 years are vulnerable to extreme low and high temperatures in lag strata 0–1 | 0-1day  2-6days  7-13days |
| Hammer et al (2006) | Nouna, Burkina Faso, | All-cause and cause-specific mortality in children and Verbal Autopsy. Poisson regression | Malaria remains the common diagnosis (42%) with the highest death rates in new-borns 6– 11 months in the rainy and warm period. | Not specified |
| Kynast-Wolf et al (2006) | Nouna, Burkina Faso | Longitudinal data from a population of ~35,000. Poisson regression, floating absolute risk method, and continuous modelling using sine functions. | Higher overall mortality during the dry season (November to May) across most age groups. For infants, the peak mortality was around the end of the rainy season | 14 -21 days |
| Sankoh et al (2003) | Nouna, Burkina Faso | Crude death and Verbal Autopsy. Poison regression | Malaria and Diarrhea accounted for 21% of total deaths in adults and 22% in older people in the dry and warm period | Not specified |
| Jankowska et al (2012) | Mali | Temperature, precipitation, livelihood, and malnutrition. Cluster analysis multivariate linear regression, and spatial correlations. | Temperature and rainfall index b−100 zone was related to stunting (β = −0.17, p b 0.00), underweight (β = −0.159, p b 0.00) and anaemia (β = −0.15, p b 0.01) for all clusters | Prolong effects on malnutrition |
| Enete et al (2013) | Douala, Cameroon | Air temperature, relative humidity, solar radiation wind speed, and global luminance. Descriptive and inferential statistics, comfort analysis, and Spearman rank correlation | Discomfort from heat stress results in stroke, cramps, exhaustion, fatigue, headache, nausea, fainting, and mortality. | Not specified |
| Dapi et al (2010) | Yaounde´ and Douala, Cameroon | Temperature, relative humidity, and surveys of schoolchildren. correlation analysis | The study found high indoor temperatures in schools affecting students' health. Symptoms like fatigue, headaches, and heat-related discomfort were common. | Not specified |
| Luque et al (2009) | Lusaka, Zambia | Temperature and Cholera. Time series, and Poisson regression | A 1.0°C rise in temperature 6 weeks before the onset of the outbreak clarified 5.2% of the rise in cholera cases (RR = 1.05, 95% CI: 1.04, 1.06). The associated hazard was 4.7% | 6 weeks |
| Jaffar et al (1997) | Upper river division, Gambia | The monthly and annual mortality rate from Post-mortem questionnaires. | 56% of mortality from acute respiratory infections, malaria, acute gastroenteritis, and septicaemia in the rainy/heat season between July-October | Not specified |
| McGregor et al (1961) | Keneba, Gambia | Monthly mortality counts. Descriptive analysis. | The high mortality rate in rainy/heat season in children above 7 years (July-October). | Not specified |
| Rayco-solon et al (2004) | Keneba, Manduar and Kantong Kunda, Gambia | Odds dying in rainy/hungry season. Cox regression and likely test model. | Seasonality in mortality with more losses in the ‘hungry’ season prior to1975 (odds ¼ 1.87; 95% CI ¼ 1.62–2.17) and from 1975 to 1984 (odds ¼ 1.84; 95% CI ¼ 1.34–2.53). | Not specified |
| Brewwster and Greenwood (1993) | Banjul, Gambia | Seasonal mortality rate. Odds ratio | Peak mortality in meningitis, pneumonia, and malnutrition in the rainy/hot season (July-December). | Not specified |
| Longo-Mbenza et al (1999) | DRC | Temperature, Haematocrit, stroke Patients admitted. Correlation and regression | Temperature was linked to haematocrit (r = 0.12, p b 0.00 and r = 0.1, p b 0.01) Temp. < 28°C increased hazard of stroke death by 21 (OR = 21; 95% CI: 15, 29) |  |
| Chang et al (2004) | Kenya, Zambia, and Zimbabwe | Temperature, Women aged 15–49 years experiencing cardiovascular events, venous thrombo-embolism, arterial stroke, and acute myocardial infarction. Time series, binomial regression, and Sensitivity analysis | 5.0°C change in *Tmean* Kenya: VTE, 0.43 (95% CI: 0.14, 1.27); stroke, 0.65 (95% CI: 0.11, 3.89) Zambia: VTE, 1.16 (95% CI: 0.21, 6.47); stroke, 0.89 (95% CI: 0.45, 1.75) Zimbabwe: VTE, 0.70 (95% CI: 0.49, 1.01); stroke, 0.91 (95% CI: 0.62, 1.34) | 1 month |
| Ng and Cowling (2014) | Guinea, Gabon, DRC, South Sudan, Uganda | Temperature, absolute humidity and reported Ebola virus disease. Time series and regression models | Cumulative ORs of EVD outbreaks related to deviations (−1, −2, and −3.0°C) from the monthly average temperature were significant with a regular dose-response relation across the lag period from 1.20 to 1.71, 1.43 to 2.93, and 1.71 to 5.00. | 2-3 months |
| Paz (2009) | Kenya, Uganda, Rwanda, Burundi, Tanzania, Zambia, Malawi, Mozambique | Air temperature, Cholera cases. Time series and Poisson regression. | A 0.1°C increase in the annual *Tmean* increases the yearly total of cholera cases by 1.87 (β = 0.63, *p* = 0.18) for recent year and 2.78 (β = 1.02, *p* = 0.03) for the preceding year. | Not specified |
| Batté et al (2018) | West Africa | Temperature and relative humidity. Pearson time correlation, ensemble, and index probabilistic forecast and the statistical–dynamical forecasting approach. | High possibilities of heatwave indices above the top quintile of 1993–2014 in utmost parts of West Africa. | Not specified |
| Pasquini et al (2020) | Dar es Salaam | Semi-structured interviews, and an engagement workshop with stakeholders. Mixed techniques. | Heat and health are currently a low-priority policy issue. Informal settlement residents have high exposure, sensitivity, and low adaptability to heat. | Not specified |
| Ermert et al (2012) | West Africa | Temperature and rainfall. Liverpool Malaria Model and integrated weather-disease model. | A declined spread of malaria for a simulated rise in temperatures and a reduction in precipitation. | Not specified |
